# Advancing psychological interventions for premenstrual dysphoric disorder: A dialectical behaviour therapy–informed treatment model

**DOI:** 10.1177/00048674251348370

**Published:** 2025-06-27

**Authors:** Aimee Oliveri, Sharryn Muir, Eveline Mu, Jayashri Kulkarni

**Affiliations:** 1 Flourishing Women Psychology, Sydney, NSW, Australia; 2 Northern Psychology Centre, Melbourne, VIC, Australia; 3 HER Centre Australia, Department of Psychiatry, School of Translational Medicine, Monash University, Melbourne, VIC, Australia

## Introduction

Premenstrual dysphoric disorder (PMDD) is a chronic mood disorder characterised by the cyclical recurrence of severe emotional and physical symptoms that arise in the 2 weeks prior to menstruation (e.g. luteal phase) and remit in the follicular phase during menstruation (American Psychiatric Association, 2013). Core symptoms include mood swings, irritability, anxiety and depressed mood; however, behavioural and somatic symptoms also occur. Diagnosis is based on the Diagnostic and Statistical Manual of Mental Disorders, Fifth Edition (*DSM*-5) and requires at least 2 months of daily symptom tracking (American Psychiatric Association, 2013).

PMDD affects 5.5% of menstruating women and, although hormonally driven, is fundamentally a neuroendocrinological condition (American Psychiatric Association, 2013). This means symptoms arise from a heightened sensitivity to normal hormonal fluctuations, particularly changes in progesterone and its metabolites (Eisenlohr-Moul, 2019). Other aetiological factors include altered serotonin function, exposure to trauma and chronic stress, with 83% of Australian women with PMDD reporting early life trauma (Kulkarni et al., 2022).

PMDD is a debilitating condition, with studies showing alarmingly high rates of comorbidity (70%), suicidal ideation (72%) and suicide attempts (34%), highlighting the need for effective interventions (Eisenlohr-Moul et al., 2022). First-line treatments for PMDD are medical, including selective serotonin reuptake inhibitors (SSRIs) and combined oral contraceptives (Eisenlohr-Moul, 2019). However, the complex nature and profound psychological impacts of PMDD necessitate a multidisciplinary approach.

Traditional cognitive behavioural therapy (CBT), which emphasises thought and behaviour modification, is the most researched psychological intervention for PMDD (Eisenlohr-Moul, 2019). Yet approaches like dialectical behaviour therapy (DBT), which emphasise acceptance and adaptive coping, may better align with the biological and chronic nature of PMDD (Linehan, 2015). Although originally developed for borderline personality disorder (BPD), DBT has since demonstrated utility in addressing emotion dysregulation, impulsivity, rejection sensitivity, interpersonal difficulties, cognitive challenges and suicidality (Linehan, 2015) – all of which are key drivers of PMDD symptom severity (Eisenlohr-Moul, 2019). Consequently, DBT may offer a more targeted treatment for women with PMDD. This does not necessarily imply a link between PMDD and BPD, but highlights DBT’s compatibility for addressing the fundamental challenges associated with PMDD.

**Figure 1.**
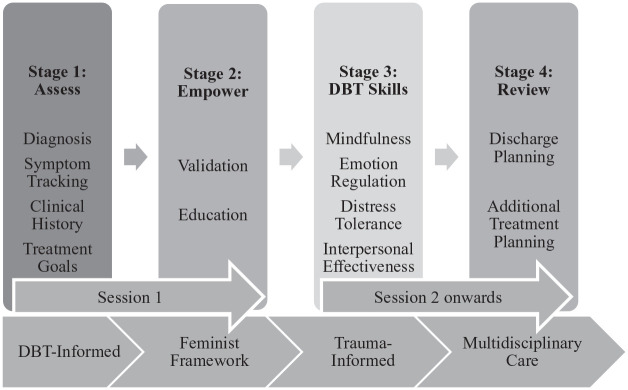
DBT-informed treatment model for PMDD.

Despite strong theoretical support for using DBT to treat PMDD, there are no formal guidelines or models for adapting DBT to PMDD. In addition, there is a lack of empirical research in this area. Drawing from existing theory and extensive clinical experience treating PMDD, this paper presents the first DBT-informed treatment model for PMDD (see Figure 1). This model aims to guide clinical practice and facilitate empirical validation, with the goal of advancing psychological interventions for PMDD.

## Theoretical model

### DBT-informed

DBT comprises four treatment modules: mindfulness, emotion regulation, distress tolerance and interpersonal effectiveness (Linehan, 2015). It can be delivered as either a standard comprehensive model or a skills-based approach, both of which have strong empirical support (Linehan, 2015). Our model uses a skill-based DBT-informed approach, focusing only on the strategies most relevant to the clients’ needs and goals. For licenced clinicians implementing this treatment model, basic DBT skills training should suffice.

### Feminist framework

Our model acknowledges the systemic and societal challenges faced by women with PMDD, including androcentric healthcare, diagnostic delays and dismissal (Kulkarni et al., 2022). Validation, empowerment and centralising the woman’s voice are therefore vital components of treatment.

### Trauma-informed

Recognising that many women with PMDD have experienced trauma, our model takes a trauma-informed approach, emphasising safety, empowerment and trauma awareness (Kulkarni et al., 2022). A phase-based approach is also used, emphasising DBT strategies to establish safety and stability.

### Multidisciplinary care

Multidisciplinary care is central to our model. We emphasise using DBT alongside medical treatments when clinically indicated, supporting optimal PMDD management. We also educate patients on treatment options, facilitate referrals and encourage collaboration. Finally, because DBT is effective in treating a range of conditions (Linehan, 2015), clinicians can address PMDD symptoms and related mental health concerns simultaneously.

## Treatment structure

Based on our experience, treatment for PMDD typically involves 10 fortnightly sessions to align with the Australian Medicare model and progresses through four stages, as shown in Figure 1.

### Stage 1: assess

Stage 1 involves gathering comprehensive information to accurately diagnose PMDD and includes:

#### Diagnosis

The client’s symptoms are assessed against *DSM*-5 diagnostic criteria for PMDD (American Psychiatric Association, 2013). The clinician should ask about the client’s menstrual cycle, symptom onset, progression and impact, while assessing for comorbidities and ruling out other explanations, such as premenstrual exacerbation (PME) of another disorder.

#### Symptom tracking

If there is enough information to support a provisional diagnosis, treatment can begin and clients are encouraged to track their symptoms for at least two cycles to establish a formal diagnosis. The daily record of severity of problems (DRSP) can facilitate tracking (American Psychiatric Association, 2013).

#### Clinical history

Clinicians should review the client’s treatment history, including past interventions, medications and contraception use. They should also assess the client’s health, family history, supports, coping strategies (adaptive and maladaptive) and suicide risk.

#### Treatment goals

Clinicians should collaborate with clients to establish realistic treatment goals. The authors use pre-treatment questionnaires (see Supplemental Materials) to identify treatment targets, such as brain fog, fatigue, irritability and low mood.

### Stage 2: empower

Stage 2 emphasises validation and psychoeducation, which, in the authors’ clinical experience, strengthen hope, self-attunement, self-compassion and self-advocacy.

#### Validation

Establish a foundation for effective treatment by creating a supportive, non-judgmental environment where women feel heard and understood. Key elements include:

- Collaborating with and recognising women as experts in their own lives.
- Restoring hope in what may feel like a hopeless condition.
- Reducing feelings of defectiveness.
- Empowering women with knowledge and tools to manage their symptoms and advocate for themselves.

#### Education

Provide comprehensive information on PMDD, covering:

- Definition, pattern and symptoms of PMDD.
- Treatment options and referral pathways.
- Aetiological components.
- Menstrual cycle awareness.
- Lifestyle adjustments, including cycle syncing and pacing.
- Resources such as the International Association for Premenstrual Disorders (IAPMD) website (Eisenlohr-Moul, 2019).

### Stage 3: DBT skills

Stage 3 introduces DBT strategies tailored to the treatment goals identified in Stage 1. Grounded in DBT principles (Linehan, 2015), Table 1 outlines the skills the authors use most often for clients with PMDD.

**Table 1.** Mapping DBT skills to PMDD symptoms.

PMDD symptoms	Corresponding DBT skill
Fatigue	Self-soothe, radical acceptance
Anger, **rage, irritability**	STOP, TIPP, check the facts
Anxiety	Identify emotion, validate emotion, cope ahead, mindfulness of current emotion, opposite action
Interpersonal **conflict**	DEARMAN, wise mind, dialectical thinking, observe, describe, effectiveness, FAST
Sadness, **emptiness**	Identify emotion, validate emotion, mindfulness of current emotion, self-soothe, radical acceptance
Negative **thought spirals**	ACCEPTS, TIPP, IMPROVE
Low **motivation**	One mindfully, opposite action

STOP = stop, take a step back, observe, proceed mindfully; TIPP = temperature, intense exercise, paced breathing, progressive muscle relaxation; DEARMAN = describe, express, assert, reinforce, mindful, appear confident, negotiate; FAST = fair, apologies (limit), stick to values, truthful; ACCEPTS = activities, contributing, comparisons, emotions, pushing away, thoughts, sensations; IMPROVE = imagery, meaning, prayer, relaxation, one thing at a time, vacation, encouragement.

Skills are introduced in sessions and practised between, particularly during low-stress periods and the follicular phase, to prepare for the luteal phase. If requested, partners or carers may attend a session, allowing clinicians to gather information, provide education, safety plan for suicidality and address relational dynamics. Progress is monitored using standardised measures (e.g. DRSP) and non-standardised measures (e.g. clinical observation).

### Stage 4: review

Clients progress to stage 4 after they have made significant progress towards their goals, developed a robust skill set and gained confidence in managing PMDD symptoms. This stage involves either discharge planning or further treatment planning.

#### Discharge planning

For clients ready to conclude therapy, the focus is on maintaining progress and managing symptoms post-treatment.

#### Additional treatment planning

For clients needing ongoing support, further treatment options are explored, such as:

- Relationship counselling.
- Trauma processing.
- Parenting support.
- Management of comorbid conditions.

## Conclusion

Our DBT-informed treatment model for PMDD offers a comprehensive approach to managing PMDD. By integrating DBT principles with feminist and trauma-informed perspectives, our model addresses the unique challenges faced by women with PMDD and represents a significant step forward in advancing psychological interventions for PMDD. While this model shows promise based on theoretical foundations and clinical experience, empirical validation is essential.

## Supplemental Material

sj-docx-1-anp-10.1177_00048674251348370 – Supplemental material for Advancing psychological interventions for premenstrual dysphoric disorder: A dialectical behaviour therapy–informed treatment model Supplemental material, sj-docx-1-anp-10.1177_00048674251348370 for Advancing psychological interventions for premenstrual dysphoric disorder: A dialectical behaviour therapy–informed treatment model by Aimee Oliveri, Sharryn Muir, Eveline Mu and Jayashri Kulkarni in Australian & New Zealand Journal of Psychiatry
