# Supplementary material for: Advancing psychological interventions for premenstrual dysphoric disorder: A dialectical behaviour therapy–informed treatment model
Source: Aust N Z J Psychiatry. 2025 Jun 27;59(8):670–3. doi: 10.1177/00048674251348370 (PMC12375139; doi:10.1177/00048674251348370)
Supplement: sj-docx-1-anp-10.1177_00048674251348370 – Supplemental material for Advancing psychological interventions for premenstrual dysphoric disorder: A dialectical behaviour therapy–informed treatment model [file sj-docx-1-anp-10.1177_00048674251348370.docx]

**PRE-PMDD TREATMENT QUESTIONNAIRE**

Please complete all the yellow sections:

| **Name** |  |
| --- | --- |
| **Date** |  |

| **What would you like to gain from seeing your Psychologist?** | ***Rating Scale:***  *0 = Not at all*  *1 = Not much*  *2 = Somewhat*  *3 = Yes, definitely*  *Please type the*  *appropriate number* |
| --- | --- |

| 1. | If I have PMDD? |  |
| --- | --- | --- |
| 2. | Why I've developed PMDD? |  |
| 3. | Information on what causes PMDD? |  |
| 4. | Skills and techniques to manage PMDD? |  |
| 5. | Information on prescription medications? |  |
| 6. | Information on complementary medications? |  |
| 7. | Information on PMDD and pregnancy? |  |
| 8. | Information on PMDD and menopause? |  |
| 9. | Information on what my partner should do? |  |
| 10. | Where to read good information on PMDD? |  |
| 11. | What else would you like your psychologist to include in your sessions? | |

1 of 2

| **Which symptom do you find the most difficult to cope with?** | ***Rating Scale:***  *0 = Not at all*  *1 = Not much*  *2 = Somewhat*  *3 = Yes, definitely*  *Please type the*  *appropriate number* |
| --- | --- |

| 1. | Rage |  |
| --- | --- | --- |
| 2. | Depressed mood |  |
| 3. | Suicidality |  |
| 4. | Irritability |  |
| 5. | Anger |  |
| 6. | Anxiety |  |
| 7. | Fatigue |  |
| 8. | Rejection sensitivity |  |
| 9. | Guilt from impact on others, ie partner, parents, children |  |
| 10. | Negative thought spirals about yourself |  |
| 11. | Wanting to leave relationship |  |
| 12. | Wanting to leave job |  |
| 13. | Self-harm |  |
| 14. | Other symptoms? | |

2 of 2
